# Correction: Half-metallic ferromagnetism and thermoelectric-efficient behavior in chalcogenide spinels MgNi_2_X_4_ (X = S, Se): a first-principles approach

**DOI:** 10.1039/d5ra90123e

**Published:** 2025-10-28

**Authors:** Ashiq Ramzan, Mudasir Younis Sofi, Mohammad Ishfaq-ul-Islam, Mohd. Shahid Khan, M. Ajmal Khan

**Affiliations:** a Department of Physics, Jamia Millia Islamia New Delhi 110025 India; b Department of Physics, Islamic University of Science and Technology Awantipora, Pulwama J&K India majkhan@jmi.ac.in

## Abstract

Correction for ‘Half-metallic ferromagnetism and thermoelectric-efficient behavior in chalcogenide spinels MgNi_2_X_4_ (X = S, Se): a first-principles approach’ by Ashiq Ramzan *et al.*, *RSC Adv.*, 2025, **15**, 24002–24018, https://doi.org/10.1039/D5RA03555D.

The authors regret that in the original submission the ORCID iD for co-author Mudasir Younis Sofi was incorrectly associated with the corresponding author, M. Ajmal Khan. The correct authorship list and affiliations are as displayed in this notice.

The Royal Society of Chemistry apologises for these errors and any consequent inconvenience to authors and readers.

